# Long-read sequencing and next-generation CRISPR editors: a unified pipeline for rare disease precision medicine with ethical and regulatory perspectives

**DOI:** 10.3389/fmed.2026.1859881

**Published:** 2026-07-06

**Authors:** Anshida Konamveettil Abdul Latheef, Mohammad Ali, Omar Farahat, Fatiha Gourari, Enas AlQodsi, Nadia Akawi

**Affiliations:** 1 Department of Genetics and Genomics, College of Medicine and Health Sciences, United Arab Emirates University, Al Ain, United Arab Emirates; 2 Faculty of Law, McGill University, Montreal, QC, Canada; 3 Department of Public Law, College of Law, United Arab Emirates University, Al Ain, United Arab Emirates; 4 Department of Private Law, College of Law, United Arab Emirates University, Al Ain, United Arab Emirates

**Keywords:** CRISPR/Cas9, ethics of gene editing, genome editing, Gulf region, long-read sequencing, rare genetic disorders

## Abstract

Rare diseases, most of which have a genetic basis, remain a major challenge due to diagnostic delays and limited therapeutic options, particularly within the Middle Eastern regions. These countries exhibit a heightened prevalence of genetic disorders attributable to their distinctive genetic architecture. Advances in long-read sequencing (LRS) technologies have significantly improved our ability to detect complex genetic variations, including structural variants (SVs), repeat expansions, and mutations in previously inaccessible genomic regions, thereby increasing the diagnostic yield in rare disease cohorts. In parallel, the rapid evolution of gene-editing platforms such as CRISPR/Cas9, base editors, and prime editors has opened new possibilities for addressing the biological pathways of the disease and achieving precise therapeutic correction of pathogenic variants causing the disease. Importantly, the integration of LRS with gene-editing approaches establishes a continuum from accurate variant discovery and functional characterization to the development of personalized therapies. This review highlights recent progress in both fields, discusses their complementary roles in rare disease research, and explores the translational opportunities and ethical challenges of combining these technologies to advance precision medicine. In addition, the review addresses emerging ethical and regulatory considerations associated with the clinical translation of long-read sequencing and next-generation gene-editing technologies, particularly in the context of rare disease precision medicine.

## Introduction

Rare diseases have emerged as a public health priority due to their collective disease burden, significant clinical burden, and frequent early onset (1). An estimated 3.5–5.9% of the global population is affected by rare diseases globally, and nearly 71.9% have an underlying genetic cause (2). For instance, populations in the Middle East exhibit a high prevalence of rare genetic diseases due to the region’s unique genetic architecture characterized by high consanguinity rates and genetically isolated populations (3–5). Studies based on Gulf countries have revealed a high frequency of autosomal recessive disorders and founder mutations, contributing to significant health challenges (Table 1) (6–16). Many rare disorders are chronic and life-threatening, and lethality often results not only from the disease itself but also from diagnostic delays and the lack of effective treatments. Consequently, a substantial gap often exists between disease onset, definitive diagnosis, and the initiation of effective therapeutic intervention.

**TABLE 1:** Prevalence, diagnostic delay, and genetic inheritance of rare diseases in gulf cooperation council (GCC) countries.

Sr. no.	Country	Rare disease	Time for diagnosis	Consanguinity status	Inheritance pattern	Prevalence (%)	References
1	UAE	Infantile-onset Pompe disease	Diagnosis often takes 0–12 months	High consanguinity rate of 25–70%	Autosomal recessive	0.0026	(7, 8)
2	Saudi Arabia	Mucopolysa- ccharidosis	Diagnosis typically takes an average of 2.6 years	High consanguinity rate of 60%	Autosomal recessive	0.0078	(9, 10)
3	Qatar	Classical homocystinuria (HCU)	Many cases remain undiagnosed for 4.3 years	Consanguinity rate of 98.4%	Autosomal recessive	0.055	(11)
4	Oman	Hirschsprung’s disease	Diagnosis commonly delayed 0–1 year	Consanguinity rate of 56.4%	Autosomal recessive or dominant	0.3	(12–14)
5	Kuwait	Primary immunodeficiency diseases	Diagnosis takes 2.05 ± 1.7 years	Consanguinity rate of 54%	Autosomal recessive	Underestimated due to lack of awareness	(15, 16)

Traditional diagnostic approaches primarily relied on clinical phenotyping and comparison with previously reported cases (17). As a result, many patients remained undiagnosed for years despite repeated hospital visits, and, in some cases, patients died without a definitive diagnosis. Accurate diagnosis is essential for proper disease management, treatment planning, and avoidance of inappropriate treatments and associated adverse effects. Moreover, in inherited rare diseases, identifying genetic variants can reveal inheritance patterns of the disease, enabling informed family planning and genetic counseling (17). The diagnostic process for rare diseases depends on the patient’s age, available resources and clinical symptoms. Patients with rare diseases often experience a diagnostic odyssey lasting more than 4–5 years, and some cases remain unsolved for over a decade (17).

The identification of molecular genetic causes has become a cornerstone of rare disease diagnostics, particularly given that approximately 80% of rare diseases have an underlying genetic basis (17, 18). These diseases may arise from a wide range of genetic factors such as single-nucleotide variants (SNVs), insertions and deletions (indels), SVs, repeat expansions, inversions, translocations, complex genomic rearrangements, and epigenetic alterations such as aberrant DNA methylation (19). Most rare genetic disorders follow Mendelian patterns of inheritance and are caused by pathogenic variants in a single gene (20). According to the 2022 Emirati dataset of the Catalog for Transmission Genetics in Arabs (CTGA), among the 665 unique conditions prevalent in the Emirati population, 621 exhibit Mendelian modes of inheritance. Remarkably, among these 665 disorders, more than half are classified as extremely rare disorders worldwide, with an autosomal recessive mode of inheritance. For instance, disorders such as Joubert syndrome 16 (OMIM #614465) and glycine encephalopathy (OMIM #605899) have been reported at disproportionately higher frequencies in the UAE compared with global populations (21).

Traditional genetic testing relied on linkage analysis and candidate gene sequencing (18). These approaches relied on tracking the inheritance of disease-associated loci within families based on inheritance pattern, and once a candidate gene was suspected, individual gene re-sequencing was performed to identify the mutation. It was a highly expensive and time-consuming process (18).

The advent of next-generation sequencing (NGS) transformed the field of rare disease diagnostics by enabling unbiased, high-throughput, and cost-effective analysis of patient genomes. Short-read sequencing (SRS) approaches, including whole-exome sequencing (WES) and whole-genome sequencing (WGS), have substantially improved the identification of pathogenic variants underlying a wide range of rare genetic disorders, including metabolic and neurodevelopmental diseases (18). However, despite these advances, a significant proportion of rare disease cases remain unsolved, partly because short-read technologies have limited ability to detect certain classes of genetic variation, including complex SVs, repeat expansions, and variants located within repetitive or highly homologous genomic regions (22). To address some of these limitations, LRS technologies have emerged as a complementary approach capable of providing more comprehensive genomic characterization and improving diagnostic yield.

Developing effective therapies remains a major challenge for rare genetic disorders (17). Despite spotting causative genes, effective treatments remain limited (17). There are very few therapies or tailored treatment plans associated with rare diseases. Platforms such as CRISPR/Cas9, prime editing and base editing offer opportunities for disease modeling, functional validation of pathogenic variants, and the development of mutation-specific therapeutic strategies. Early diagnosis and timely therapeutic intervention are particularly important for disorders associated with significant morbidity or mortality (23).

Here, we propose that the integration of advanced technologies, such as LRS and CRISPR/Cas9–mediated genome editing, represents a promising strategy to accelerate diagnostic workflows and enable the development of mutation-specific therapeutic interventions. Nevertheless, region-specific ethical, legal, and regulatory considerations related to the clinical translation of these technologies in humans must be carefully evaluated and addressed. Literature was identified through PubMed, Google Scholar and Scopus using terms such as “rare diseases Gulf region,” “rare genetic disorders,” “long-read sequencing,” “next-generation sequencing,” “CRISPR gene editing,” “prime editing,” “base editing,” “ethical and legal aspects of gene editing,” and “Islamic Ethical Foundations.” No formal systematic review protocol was applied; studies were selected based on direct relevance to the manuscript’s themes.

## Long-read sequencing as a diagnostic technology

NGS technologies have generated a remarkable leap in the research of rare diseases (Table 2) (24). The advent of NGS technologies enabled the widespread adoption of SRS approaches, WES and WGS. These tools have aided in understanding the genetic etiology of numerous disorders which were undetectable by the earlier testing methods. WES rapidly became a widely adopted diagnostic tool because of its cost-effectiveness and its ability to sequence the protein-coding regions of the genome. Although exons comprise < 2% of the human genome, they harbor nearly 85% of large-effect disease-causing variants (24). In contrast, the WGS method sequences the whole genome using short reads, expanding the diagnostic capability and improving the detection of certain regulatory and intronic variants that are typically not captured by WES (22). Consequently, patients who remain undiagnosed following WES are frequently referred for WGS, which can provide an additional diagnostic yield through more comprehensive variant detection (17).

**TABLE 2:** Key technical differences across widely known next-generation PLATFORMS.

Platform	Core principle	Amplification	Read length	Strengths	Limitations	References
Illumina (sequencing-by-synthesis)	Reversible terminator nucleotides incorporated one base at a time with imaging cycles.	Yes—bridge amplification on flow cell.	75–600 bp.	High accuracy, high throughput.	Short reads limit structural variant detection.	(28)
Ion torrent	pH change detection from released hydrogen ions during DNA synthesis.	Yes—emulsion PCR.	200–400 bp.	Fast and lower cost.	Homopolymer errors.	(28)
BGI/MGI DNBseq	Rolling circle amplification-based DNA nanoball formation.	Yes—RCA.	100–150 bp PE.	Low duplication; reduced index hopping.	Short reads; platform availability.	(28)
PacBio SMRT	Real-time fluorescent nucleotide incorporation in Zero-Mode Waveguides.	No	10–25 kb; HiFi reads > 99.8%.	Precise long reads; excellent for SVs and isoform detection.	Higher sequencing cost.	(27, 30)
Oxford nanopore (ONT)	Ionic current disruption as DNA passes through nanopores.	No	5 kb to > 100 kb.	Longest reads; portable; real-time.	Higher raw error rates.	(27, 29)

Nevertheless, the SRS method still fails to detect a substantial proportion of pathogenic genomic variations, leaving nearly half of the patients with a suspected Mendelian disorder undiagnosed (20). SRS methods are particularly effective for detecting SNVs and small insertions and deletions (indels) (25, 26). The SRS method is limited in detecting large insertions or deletions, copy number abnormalities, non-exonic splice alterations, or large rearrangements (22). It has read lengths varying between 50 and 300 bp, which inherently limits the assembly of repetitive elements, detection of GC-rich regions, and identification of complex structural regions (27). Furthermore, SRS struggles with *de novo* genome assembly, haplotype phasing, detection of transcript isoforms, precise mapping of SVs and direct detection of DNA methylation (28). These limitations underscore the need for advanced technologies capable of capturing long-range genomic information (26, 29).

LRS can provide read lengths ranging from 1,000 to over 1 million base pairs (30). This enables high-resolution detection of SVs, repeat expansions, DNA methylation, and insights into complex genomic regions that were not readily resolved by conventional methods. Diseases that remain undiagnosed following conventional genetic testing may be further investigated using LRS, which offers improved detection of complex genetic variants, particularly SVs that are frequently missed by other methods (20). LRS can determine the haplotype phase, revealing the inheritance pattern of the proband’s mutation, which is essential, especially in cases of compound heterozygosity and autosomal dominant conditions (20).

PacBio and Oxford Nanopore (ONT) are two of the LRS platforms designed to widely used to resolve complex genomic regions (Table 2) (31). PacBio uses the single-molecule real-time (SMRT) sequencing method while ONT utilizes the nanopore-based sequencing method. PacBio HiFi typically produces reads of ∼25 kb with single-molecule consensus read accuracy of ≈99.8–99.9% (32). In contrast, ONT generally produces longer reads, with N50 values often in the 20–40 kb range and ultra-long reads exceeding 100 kb. However, sequencing accuracy is highly dependent on sequencing chemistry and base-calling algorithms, with recent R10.4.1 chemistries achieving raw read error rates of approximately 1% under optimized conditions (31). Additionally, both ONT and PacBio platforms can directly detect cytosine base modifications, including 5-methylcytosine (5mC) and 5-hydroxymethylcytosine (5hmC), thereby enabling simultaneous identification of pathogenic epigenetic alterations alongside genomic variants (33).

Recent years have witnessed a substantial expansion in the use of genomic technologies across Gulf countries (34–37). These countries have begun to utilize NGS across clinical diagnostics, population sequencing, and research. Programs such as population-scale WGS and clinical WES are already producing region-specific variant catalogs and improving diagnostic yields (35, 36). However, only a small number of research and diagnostic initiatives currently incorporate long-read technologies (34–37).

Collectively, studies (19, 26, 38–44) comparing LRS and SRS demonstrate that LRS provides substantial advantages for the detection of structural variants, repeat expansions, methylation signatures, and variants located within complex genomic regions, particularly in cases that remain unresolved following conventional short-read sequencing (Table 3). Moreover, the direct methylation detection property of LRS represents a distinct advantage of LRS as it enables comprehensive epigenetic profiling that is critical for the accurate clinical diagnosis of certain conditions, such as cancer and neurodegenerative disorders (33). Although LRS provides additional diagnostic yield, more studies focusing solely on LRS are required to fully assess its standalone diagnostic potential. Continued efforts to reduce the cost, improve accessibility, enhance sequencing accuracy, and establish standardized analytical pipelines will be essential for large-scale adoption and optimal clinical utility of LRS (19, 20).

**TABLE 3:** Comparative diagnostic outcomes of short-read sequencing (SRS) and long-read sequencing (LRS) in rare and undiagnosed genetic disorders.

Disease/condition	SRS findings	LRS findings	Remarks	References
Autism spectrum disorder (ASD)—Emirati family	Detected 509 small-size SVs; limited detection of non-coding variants	Identified 987 SVs (1–100 kb), including variants affecting non-coding regions and ncRNAs	Demonstrated superior SV detection capacity of LRS over SRS, especially for non-coding regulatory regions relevant to complex disorders like ASD	(38)
Carney complex	No pathogenic SNVs or indels detected; unable to detect large indels > 50 bp	Identified a De novo deletion removing first coding exon of PRKAR1A	LRS identified the causal variant missed by SRS due to size limitations of short reads	(26)
Glycogen storage disease type 1A (GSD1A)	WES detected a false homozygous point mutation in G6PC; inheritance inconsistent with father	ONT-LRS detected a novel deletion in one allele and a missense variant on the other allele of G6PC	LRS resolved compound heterozygosity, enabling successful PGD and IVF for selection of a healthy embryo	(39)
Undiagnosed neurodevelopmental disorders (6 children)	No causative variants identified	PacBio-LRS detected medically relevant variants in 2 out of 6 cases	Trio-based LRS identified diagnosis for 2 exome-negative neurodevelopmental cases	(40)
Thalassemia	SRS detected common variants only	PacBio-LRS detected 10 rare clinically relevant variants (3 SVs, 7 SNVs, including one novel SNV)	LRS complemented conventional testing; all rare variants validated by PCR and Sanger sequencing	(41, 42)
Structural variation–associated disorders (CNVs, repeat expansions, translocations)	Identified previously known variants but with limited breakpoint resolution	Targeted LRS confirmed all known variants and refined breakpoint location	LRS provided 100% detection with higher structural resolution	(19)
Familial cortical myoclonic tremor with epilepsy (FCMTE1)	WES and WGS reports negative	PacBio and ONT-LRS identified intronic repeat expansion in SAMD12	LRS enabled detection of pathogenic repeat expansion missed by conventional sequencing in Chinese Population	(43)
Inherited retinal dystrophy (IRD) in 3 patients	NGS report negative	LRS identified pathogenic variants in all 3 patients	LRS resolved genetically heterogeneous IRD cases previously undiagnosed by NGS	(44)

Despite the promising potential of LRS, several challenges continue to limit its widespread adoption in clinical practice. Although sequencing accuracy has improved substantially with technical advances in PacBio HiFi and ONT platforms, further standardization of laboratory protocols, bioinformatics pipelines, quality-control metrics, and variant-interpretation frameworks is required before LRS can be routinely adopted across clinical diagnostic laboratories. In addition, the analysis of LRS data remains computationally complex, and there is limited availability of validated clinical-grade tools for the detection, visualization, and interpretation of certain variant classes, particularly structural variants, short tandem repeat expansions, and methylation signatures. Practical barriers also include the need for high-molecular-weight DNA extraction, higher sequencing costs compared with SRS, lower throughput, and specialized computational infrastructure and expertise. Furthermore, comprehensive long-read allele-frequency databases and fully validated interpretation workflows are still under development, which may complicate the interpretation of variants identified in previously inaccessible genomic regions (45). Although several genetic centers have initiated pilot studies and early clinical implementation of LRS, broader integration as a routine first-line diagnostic test will require additional clinical validation studies, workflow standardization, health-economic evaluation, and continued advances in both laboratory and bioinformatic methodologies.

## Gene editing as a therapeutic strategy

Diagnosis alone is insufficient for effective disease management, particularly in the context of rare disorders (46). Early therapeutic intervention is particularly important for disorders associated with progressive mortality. The development of effective therapeutic interventions remains a major challenge. Unlike common diseases, rare disease management often requires personalized treatment strategies rather than the use of standard, widely available medications. Molecular diagnosis provides insight into the underlying genetic basis of disease and may create opportunities for the application of genome-editing technologies. CRISPR-based tools can be integrated downstream of comprehensive genomic sequencing to facilitate functional validation, disease modeling, and the development of mutation-specific therapeutic strategies (46).

## Genome editing tools

Programmable genome-editing technologies began to emerge in the late 1990s and early 2000s, transforming genetic research and functional genomics (47). Both simple and intricate genomes were manipulated using certain techniques such as transcription activator-like effector nuclease (TALEN), zinc finger nuclease (ZFN), and CRISPR (Table 4). TALEN and ZFN were widely used for more than a decade (48–51). However, their broader application was limited by technical complexity, relatively low editing efficiencies, reduced specificity, and challenges in large-scale implementation (48–51). In contrast, CRISPR rapidly emerged as one of the most powerful genome-editing tools, offering advantages in cost, efficiency, speed, and overall simplicity (47). Recent advances in gene editing have introduced RNA-guided bridge recombinases as a powerful tool beyond existing CRISPR/Cas9 methodologies. This approach enables precise insertions, deletions, and inversions of large DNA fragments at disease-relevant genomic loci, making it a promising strategy for correcting pathogenic structural variations (52) (Table 4).

**TABLE 4:** Comparison between different genome editing tools.

Feature	Meganucleases	Zinc finger nucleases (ZFNs)	TAL effector nucleases (TALENs)	CRISPR/Cas systems	RNA-guided bridge recombinases (ISCro4)
Recognition mechanism	Natural protein-DNA recognition; targets long, specific DNA motifs.	Modular zinc-finger proteins recognize 3-bp motifs; engineered for each target site.	TALE repeat arrays recognize single nucleotides; customizable DNA-binding domains.	RNA-guided recognition via complementary sgRNA.	RNA-guided recognition via a bipartite bridge RNA with two programmable loops — Target-Binding Loop (TBL) and Donor-Binding Loop (DBL)
Ease of design	Very difficult to re-engineer; limited target flexibility.	Technically challenging—requires protein engineering for each new target.	Easier than ZFNs but still requires protein assembly of long TALE arrays.	Very easy; only sgRNA sequence needs to be redesigned.	Moderately easy; both TBL and DBL sequences need to be reprogrammed
Specificity	Very high due to long recognition sequences.	High, but off-target risk if zinc fingers mispair.	High specificity due to long recognition domains.	High specificity, PAM-dependent; improved with engineered Cas variants.	Moderate specificity due to short 14-nucleotide recognition site
Editing efficiency	Low to moderate; constrained by engineering difficulty.	Moderate; varies depending on zinc-finger quality.	High; generally, more efficient than ZFNs.	High efficiency across organisms and cell types.	Moderate to good; deletions and inversions > 10%, insertions at 4–6% at endogenous loci
Multiplexing ability	Very low; typically, one modification at a time.	Limited; requires multiple engineered proteins for multiple loci.	Limited; each target needs its own TALEN pair.	Strong; multiple sgRNAs can target several loci simultaneously.	Not yet explicitly explored
Cost and accessibility	High cost; complex engineering.	High cost; proprietary designs previously restricted access.	Moderate to high cost; labor-intensive protein construction.	Low cost; widely accessible and easy to implement.	Not explored much, compact size and mRNA + synthetic oligonucleotide delivery suggest cost effectiveness
Delivery constraints	Small proteins but limited targeting flexibility.	Compact size allows vector delivery; however, optimisation required.	Large protein size complicates viral delivery.	Multiple Cas variants (e.g., Cas9, Cas12, CasΦ) allow flexible delivery.	mRNA electroporation with synthetic modified oligonucleotides without viral vectors
Applications	Rarely used today; niche genome-editing applications.	Gene knockout, targeted mutagenesis, early genome- editing studies.	Gene editing, crop engineering, functional genomics.	Broadest use: gene editing, base editing, prime editing, epigenetic control, diagnostics.	Scar-free deletions, inversions, and large fragment insertions; correction of SVs, suitable for HDR-deficient cells

## Methodology and application of CRISPR

**FIGURE 1:**
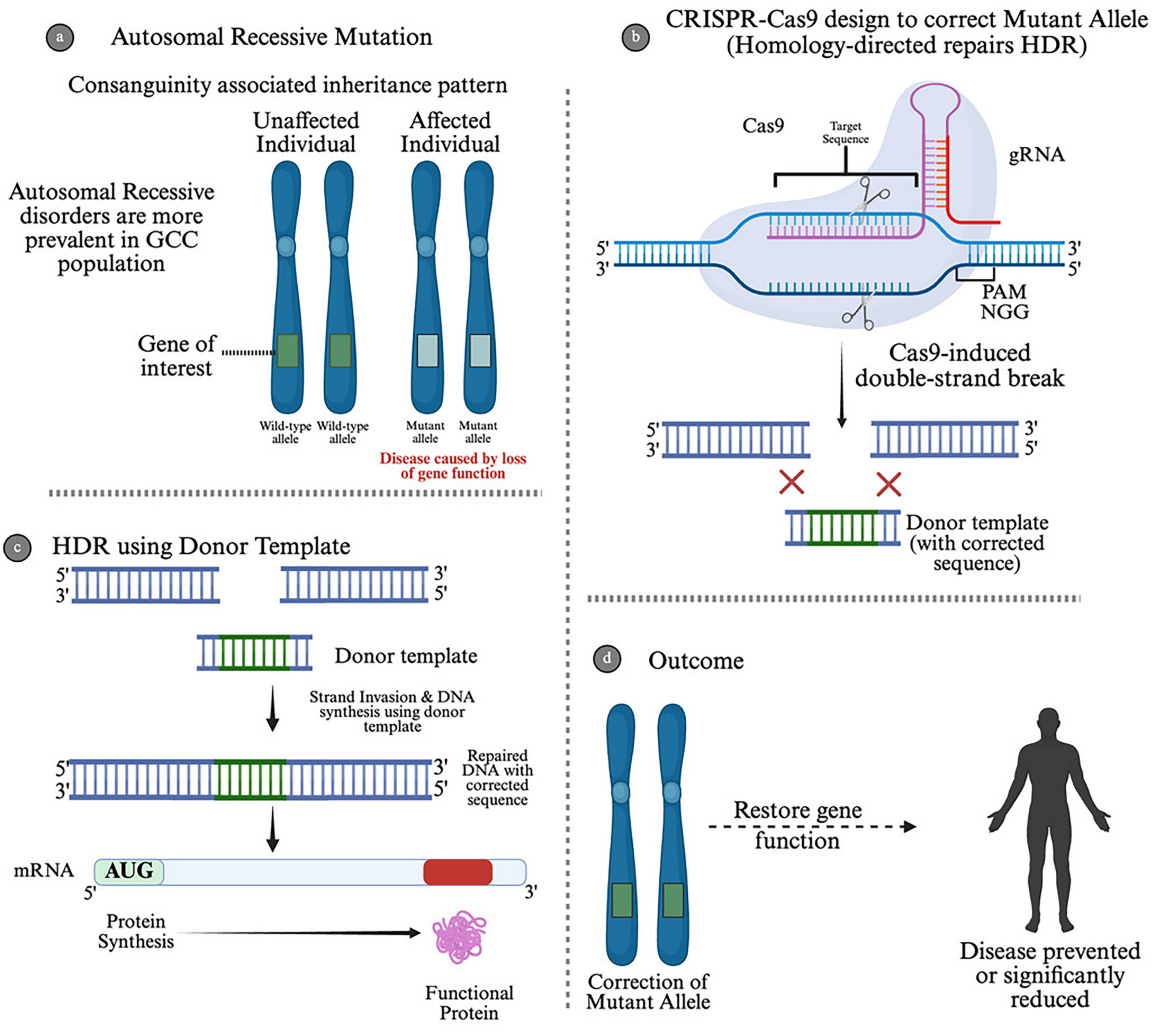
CRISPR/Cas9-based correction of autosomal recessive mutation. **(a)** Schematic representation of autosomal recessive inheritance patterns commonly associated with consanguinity in GCC populations. Individuals carrying two mutant alleles exhibit loss-of-function mutations leading to disease manifestation, whereas individuals carrying wild-type alleles remain unaffected. **(b)** CRISPR–Cas9-mediated genome editing strategy for targeted correction of the mutant allele. Cas9, guided by a gRNA, recognizes the target DNA sequence adjacent to the PAM site and induces a double-strand break. A donor DNA template containing the corrected sequence is supplied to facilitate homology-directed repair (HDR). **(c)** HDR-mediated repair process using the donor template to restore the normal DNA sequence. Corrected genomic DNA enables proper transcription and translation, resulting in the production of a functional protein. **(d)** Therapeutic outcome following successful correction of the mutant allele, leading to restoration of gene function and potential prevention or reduction of disease severity. Created with BioRender.com.

CRISPR was originally identified as an adaptive immune system in bacteria and archaea that provides protection against invading bacteriophages and foreign genetic elements. Researchers manipulated this natural system to create targeted incisions in DNA for various research and clinical purposes (46). The CRISPR/Cas9 system consists of 2 key components: a Cas9 protein that functions as a an endonuclease to introduce double-stranded breaks (DSB) at specific locations and a guide RNA (gRNA) which directs Cas9 to the targeted site. Target recognition requires the presence of a protospacer adjacent motif (PAM), after which Cas9 introduces a double-stranded DNA break at a defined genomic location (47). Among the various CRISPR-associated nucleases, Streptococcus pyogenes Cas9 (SpCas9) remains one of the most widely used because of its efficiency, versatility, and ease of implementation. Following DNA cleavage, the break is repaired through endogenous cellular repair pathways, primarily non-homologous end joining (NHEJ) and homology-directed repair (HDR) (Figure 1) (53). NHEJ typically results in gene disruption, whereas HDR can be exploited to introduce precise genetic modifications by insertion or deletion of desired nucleotides (Figure 1). These features make CRISPR a powerful and versatile tool for genome editing (46).

CRISPR-based genome-editing tools are relatively straightforward to implement and can achieve high editing efficiencies in many experimental settings; however, concerns about off-target effects, unintended genomic alterations, and delivery constraints continue to pose significant challenges to clinical translation (46). These tools facilitate functional validation of pathogenic variants, investigation of disease mechanisms, and development of targeted therapeutic strategies. CRISPR platforms are versatile with wide-ranging applications in humans, animals and plant systems. Moreover, their clinical potential has become increasingly evident with several therapeutic applications demonstrated over the past decade. Rapid advances in CRISPR research have led to multiple successful preclinical studies and an increasing number of clinical trials (Table 5) (46, 54–57).

**TABLE 5:** Clinical applications of CRISPR/Cas9–based therapeutic strategies for the treatment of inherited genetic disorders.

Disease/condition	Target gene/regulatory element	CRISPR strategy used	Therapeutic outcome	Remarks	References
Sickle cell disease (SCD) and β-thalassemia	BCL11A silences the fetal Hb production (target is to disrupt BCL11A binding sites)	*Ex vivo* CRISPR/Cas9 editing of autologous haematopoietic stem cells and progenitor cells (HSPCs)	∼80% Edited alleles at target locus after 1 year; increased γ-globin and fetal hemoglobin levels; sustained clinical improvement	Disruption of BCL11A enhancer reactivates fetal hemoglobin expression; no detectable off-target effects reported	(54)
Inherited retinal degeneration	Disease-causing retinal gene(s) (targeted *in situ*)	*In vivo* CRISPR/Cas9 delivered via subretinal injection	Improvement in visual function observed in treated patients	First-in-human CRISPR therapy for retinal degeneration; larger cohorts required to establish long-term safety and efficacy	(56)
hATTR (hereditary transthyretin amyloidosis	TTR gene	*In vivo* TTR gene editing through intravenous injection using therapeutic agent NTLA-2001 intended to knock-out TTR gene	Dose-dependent reduction in circulating TTR levels (with an average of 52 and 87% TTR reduction in the lower-dose group and the higher-dose group, respectively) No serious adverse events were found.	Phase I clinical trial in six hATTR patients with polyneuropathy received NTLA-2001 (Lipo neucleo proteins encapsulate Cas9 mRNA and a sgRNA targeting the TTR gene) injection	(55)
Relapsed/refractory CD19-positive B-cell acute lymphoblastic leukemia (B-ALL)	*TRAC* (T-cell receptor alpha chain): disrupted to prevent graft-versus-host disease and CD52 Removed to provide infused cell survival advantage	Multiplexed CRISPR/Cas9 knockout of *TRAC* and *CD52*, with *CAR19* transgene expression linked to *TRAC* disruption via a self-inactivating lentiviral vector; | | Cas9 *m*RNA delivered by electroporation on an automated CliniMACS Prodigy platform;	6 Children infused with TT52CAR19 cells; 4/6 showed *in vivo* cell expansion; 4/6 achieved flow cytometric remission and MRD negativity at day 28; all 4 responders proceeded to allogeneic stem cell transplantation	Negligible off-target activity confirmed by NGS: mechanism of non-expansion in 2 patients not identified	(96)

Despite these advances, clinically translatable gene-editing studies originating from GCC populations remain limited. Although a small number of proof-of-concept and early-stage investigations have been reported, the translation of gene-editing technologies into clinical practice within the region remains at an early stage (58).

## Limitations of early CRISPR

Significant advances have been made in CRISPR-based genome-editing technologies over the past decade (59). HDR-mediated CRISPR editing enables the precise insertion, deletion, or correction of specific DNA sequences at target loci. However, HDR-based approaches are often limited by low editing efficiency and the potential generation of unintended insertions or deletions (indels), which may compromise the intended genetic correction (59). Furthermore, because HDR depends on the cellular homologous recombination machinery, its application is largely restricted to actively dividing cells, thereby limiting its therapeutic utility for many genetic disorders (50). To overcome these limitations, next-generation genome-editing technologies, including base editing and prime editing, have been developed using the fundamental principles of the CRISPR/Cas9 system (59). Unlike conventional CRISPR/Cas9 editing, which relies on the generation of DSBs, base editing and prime editing enable precise nucleotide modifications without introducing DSBs, thereby reducing the frequency of undesired indels and potentially improving editing precision and safety (59).

Genome editing tools play a vital role in the investigation of novel therapeutic strategies for rare genetic diseases (60). Genome integrity remains a central concern during genome editing, as off-target cleavage, unintended large deletions, and chromosomal rearrangements are known risks, particularly for DSB-based approaches. Base and prime editors substantially reduce these risks but still require comprehensive off-target analyses. Long-term follow-up studies are essential to assess durability, immune responses, and potential oncogenicity. Current CRISPR-mediated methods emphasize precision, efficiency and specificity to broaden the clinical translation and application scope of genome editing (60).

## Base editing

Base editing allows the installation of direct point mutations. This method is advantageous over the conventional CRISPR approach in editing large genes (61), and it can also be applied to autosomal dominant disorders (59). There are two types of base editors, cytosine base editors (CBEs) and adenine base editors (ABEs). These editors mediate single–base transitions by converting CG to TA (CBEs) or AT to GC (ABEs) (59). Further research has focused on improving their efficiency, expanding the target range and enhancing their specificity to minimize off-target effects (61).

**FIGURE 2:**
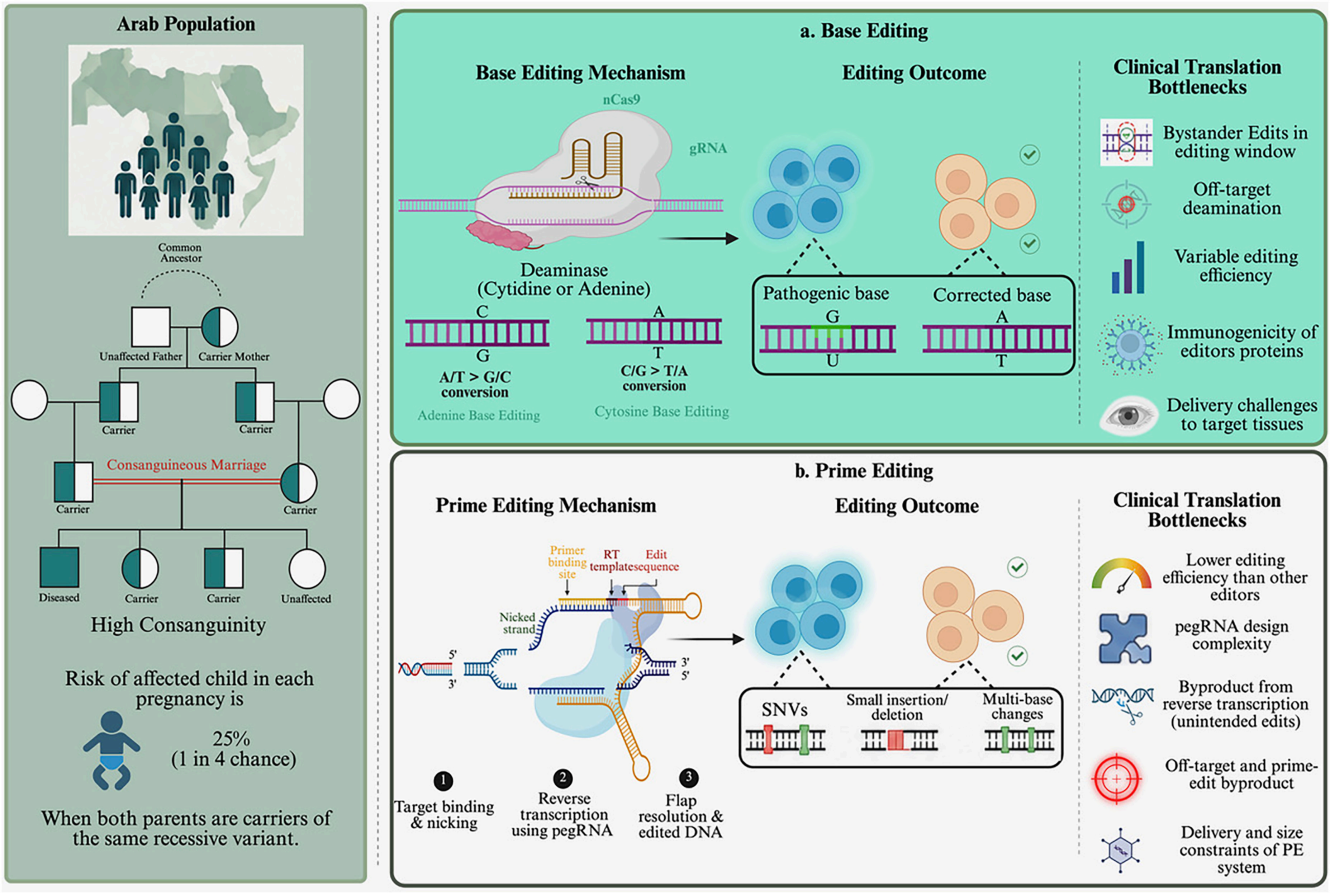
Base and prime editing strategies for rare genetic disorders: mechanisms and translational challenges. Schematic overview of base editing and prime editing strategies in the context of Arab populations. The left panel outlines population consanguinity and the inheritance pattern of rare diseases prevalent in the Arab Population. The central panel presents two parallel genome editing approaches: the upper row depicts base editing, while the lower row illustrates prime editing. Base editing utilizes a Cas9 nickase (nCas9) fused to a deaminase enzyme, guided by a single-guide RNA (sgRNA), enabling precise C∙T (cytosine base editors, CBEs) or A∙G (adenine base editors, ABEs) conversions without inducing double-strand breaks. Prime editing employs an nCas9 fused to a reverse transcriptase and a prime editing guide RNA (pegRNA) that encodes the desired edit. The editing process involves target binding and nicking, reverse transcription of the edited sequence, and flap resolution, allowing precise installation of small-sized corrections without double-strand breaks. The right panel summarizes the key clinical translation bottlenecks associated with each strategy. Created with BioRender.com.

Most advanced base editors consist of two functional components. First, there is a modified Cas9, typically, Cas9 nickase (nCas9), which creates a single-strand cut at the target location instead of a full DSB (59). Secondly, a deaminase domain that chemically alters the targeted nucleotide. In CBEs (Figure 2), the deaminase converts cytosine to uracil, whereas in ABEs (Figure 2), adenine is converted to inosine. During DNA replication, uracil is interpreted as thymine and inosine as guanine, thereby achieving a precise and predictable base substitution at the intended locus. Base editors represent promising advancements in the genome editing field, broadening therapeutic applications by providing more efficient and highly accurate genetic correction (59). However, base-editing technologies still face several challenges (Figure 2).

A relevant study on vanishing white matter (VWM) disease used an adenine base editor to correct a disease-causing *Eif2b5* mutation directly in the brains of newborn mice. A remarkable neurological editing rate of 46% was achieved, which was exceptionally high considering the neurological location (62). Another important advance came from a study on Duchenne muscular dystrophy (DMD), in which investigators used an ABE to correct 12 different nonsense mutations in separate human induced pluripotent stem cell (iPSC) models derived from 12 individual DMD patients. The efficiency of correcting the pathogenic variants varied widely across these mutations, with some achieving editing rates up to 80% (63). Recently, researchers applied base editing to slow the harmful repeat expansions that drive Huntington’s disease and Friedreich’s ataxia by manipulating DNA repair pathways (64). A Base editing strategy was recently applied clinically to treat SCD patients. This method was effective in increasing the non-sickle fetal Hb and decreasing sickle Hb, supporting further investigation of this method as a potential treatment for SCD (65). A major milestone in precision genome editing was achieved in 2025 with the first personalized *in vivo* base-editing therapy. A 7-month-old infant with severe carbamoyl phosphate synthetase 1 (CPS1) deficiency was treated using a bespoke lipid nanoparticle-delivered base editor tailored to the patient’s pathogenic variant. Given that severe CPS1 deficiency is associated with mortality rates approaching 50% during early infancy, this case highlights the potential of individualized genome-editing therapies for life-threatening rare genetic disorders (66). Collectively, these studies highlight not only the rapid technical progression of base editing, but also its growing capacity to address the complex genetic mechanisms underlying rare diseases, particularly in geographically isolated and highly inbred locations such as the Gulf region (Figure 2).

However, base-editing technologies continue to face several limitations, including bystander editing within the target editing window, off-target deamination events, and variable editing efficiencies across different genomic loci. Additional challenges include the potential immunogenicity of editor proteins, difficulties in achieving efficient and tissue-specific delivery, and a restricted editing scope. In particular, base editors are primarily limited to single-nucleotide substitutions and are generally unsuitable for introducing larger insertions, deletions, or more complex genomic modifications (59) (Figure 2). Although several base-editing therapies have entered early-stage clinical trials, broader clinical translation will require further optimization of delivery platforms, comprehensive safety evaluations, scalable manufacturing processes, regulatory assessment, and long-term follow-up studies to evaluate the durability and safety of editing outcomes (67–69). Consequently, while base editing demonstrates considerable therapeutic potential, its routine clinical application for most rare genetic diseases will likely require continued technological refinement, extensive clinical validation, and long-term safety monitoring.

## Prime editing

Prime editing is a next-generation genome editing tool, and theoretically, up to 89% of all known pathogenic mutations can be resolved using this approach (70). Its key advantage over other methods lies in its ability to expand the editable mutation spectrum to all 12 possible transitions or transversions, as well as to introduce small insertions and deletions with high precision (70). Main components of the prime-editing system consist of a modified nCas9 fused to a reverse transcriptase enzyme and a gRNA specifically designed for prime editing called pegRNA. The pegRNA contains a reverse transcription template encoding the desired edit, a prime binding site that initiates reverse transcriptase activity, and the standard target sequence that directs the nCas9 to the target site (70) (Figure 2).

A recent prime editing study (71) introduced a quadruple pegRNA strategy (QuadPE) for efficient and programmable insertion of gene-size DNA fragments. It bypasses the limitation of existing prime editing by efficiently inserting more than 300 bp (ranging from 1.6 to 26 kb) at desired genomic loci with an efficiency of more than 60%. This method utilizes four strategically coordinated pegRNAs. One pair aligns with the genomic integration site, and the other guides a DNA donor, facilitating reverse transcriptase (RT)-mediated gap-filling and nuclear delivery of large donor DNA (71).

Numerous preclinical trials have been conducted since the advent of prime editing, demonstrating its growing potential and success across animal models and cell lines. Recent studies (72, 73) have successfully applied prime editing to modify genetic material in the mouse brain, heart and liver. These investigations not only achieved accurate correction of the targeted mutation but also demonstrated minimal off-target effect and negligible toxicity. Importantly, these studies provided evidence that prime editing can operate effectively within neurologically relevant and highly complex organs such as the brain. A clinically relevant first-in-human prime-editing program for Chronic Granulomatous Disease is currently underway (74). Initial patient data indicate positive progress with minimal off-target effects, thereby establishing a strong foundation for future clinical applications (74). Given the high rates of consanguinity and the increased prevalence of rare genetic disorders in GCC populations, prime editing represents a promising and potentially transformative therapeutic strategy (Figure 2).

Although prime editing represents one of the most advanced CRISPR-based genome-editing technologies, it continues to face several limitations, including variable editing efficiency, pegRNA design complexity, unintended editing byproducts, and a limited capacity for introducing large DNA insertions (75) (Figure 2). The large size of prime editor constructs presents a significant delivery challenge, particularly for *in vivo* applications. While approaches such as split-editor systems, lipid nanoparticles, and ex vivo delivery strategies are being actively developed, achieving efficient and tissue-specific delivery remains a major obstacle to clinical translation. Additional concerns include off-target editing, potential immunogenicity, variability in editing performance across different cell types, and the need to establish the long-term safety and durability of therapeutic benefits (75). Further advances in delivery technologies, editing efficiency, safety assessment, and clinical validation will be essential to support the broader clinical translation of prime-editing therapies.

While genome editing technologies hold considerable promise for advancing personalized medicine, their wider clinical applicability requires careful and critical evaluation, particularly in conditions were established, cost-effective pharmacological therapies already exist. For instance, a recently published study utilized *in vivo* base editing gene therapy for the treatment of heterozygous familial hypercholesterolemia (HeFH) (76). Although this approach offers the advantage of eliminating repeated drug administration, it necessitates long-term patient monitoring and raises concerns of off-target effects and long-term safety. In Contrast, the majority of HeFH cases can be effectively managed through conventional lipid-lowering therapies, including statins, ezetimibe, and specific monoclonal antibodies (76). Similar gene editing studies are being conducted for diseases like hereditary thrombophilia (77), where medications such as heparin and warfarin are available to achieve ideal therapeutic outcomes (78). Ultimately, the choice of treatment modality is influenced not only by clinical factors but also by patient preference and their socioeconomic circumstances. Notably, in the context of rare conditions, particularly those prevalent in isolated populations like in the Gulf region, where conventional pharmacological treatments remain largely unavailable, gene therapy emerges as one of the most reliable and clinically compelling therapeutic avenues, underscoring its transformative potential in these populations.

## Genome editing delivery agents

One of the major challenges associated with CRISPR-based editing is the effective and targeted delivery of the gene editing system (79). Development of safe, immunologically less responsive, and efficient delivery platforms is crucial for a meaningful therapeutic output. Ex vivo delivery of editing components into cultured mammalian cells can be achieved through methods such as electroporation, viruses and lipid-based transfection. For *in vivo* applications, viral vectors remain the most widely used delivery systems. Adenovirus, lentivirus, and adeno-associated virus (AAV) are commonly employed, as they can infect both dividing and non-dividing cells (79). Lentiviral vectors with a packaging capacity of approximately 8.5 kb are used in a variety of target tissues, particularly in mice. Although AAV vectors exhibit comparatively lower packaging capacity, numerous engineered AAV variants have been developed to target specific cell types with high delivery efficiency and reduced immunological response. Additionally, emerging technologies such as engineered viral-like particles and compact Cas variants provide promising strategies to improve delivery precision and limit immunogenicity (79).

## Long-read sequencing-guided CRISPR pipeline

**FIGURE 3:**
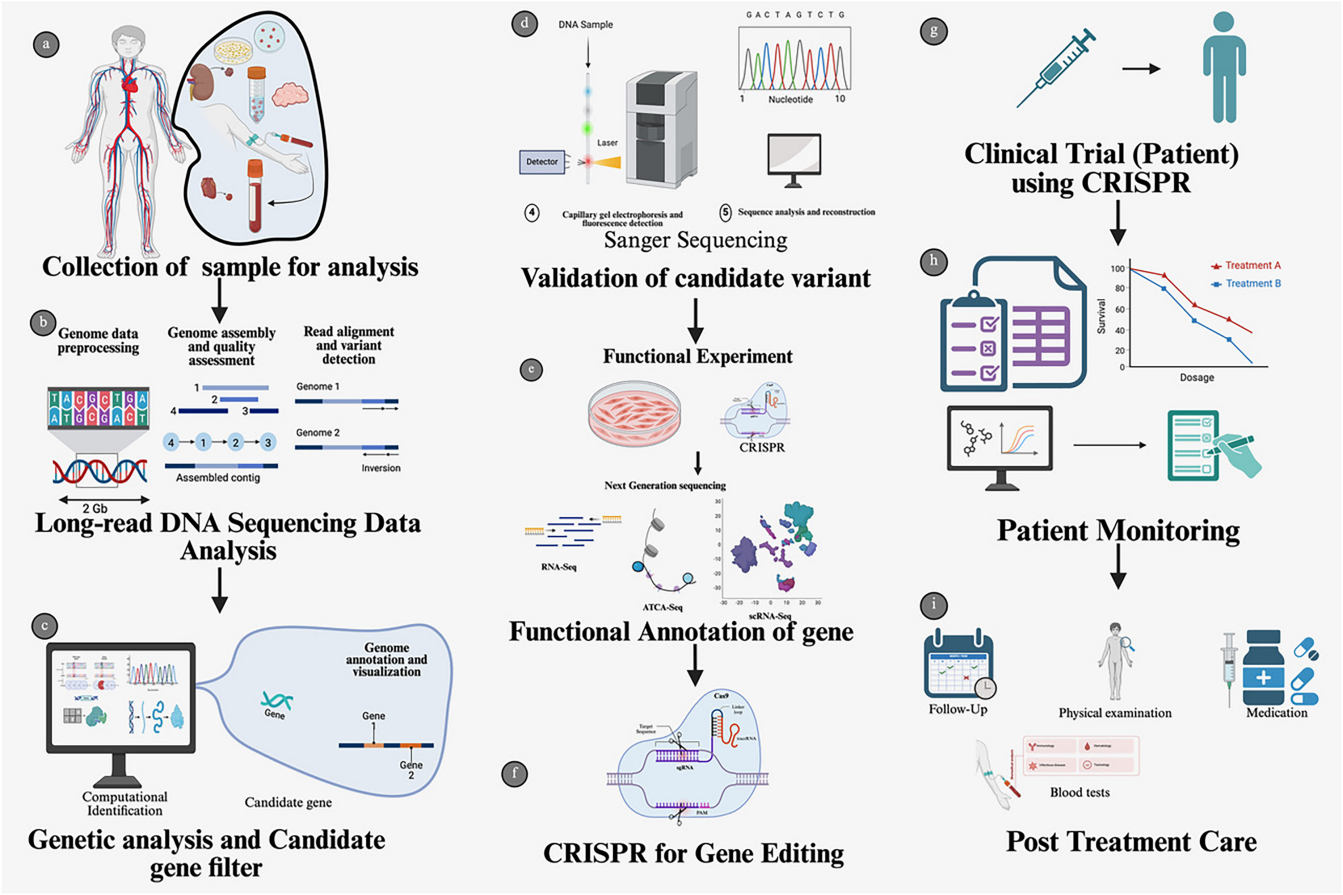
LRS-guided CRISPR pipelines. The figure depicts a complete flow chart from patient sample collection to personalized treatment using LRS-guided CRISPR pipeline. **(a)** Collection of patient samples for primary clinical testing, **(b)** perform LRS as the primary analysis, **(c)** computational analysis of LRS data to identify the candidate variant, **(d)** once a candidate variant is suspected, sanger sequencing to confirm the presence of mutation, **(e)** functional experiments using CRISPR and next generation sequencing tools to functionally annotate the gene or variant, **(f)** performing CRISPR technique using cell lines and different humanized animal models, to identify the most efficient and minimal off target treatment combination, **(g)** incorporation of the treatment combination in the patient, **(h)** monitoring the patient closely to validate the effect of treatment, and **(i)** post treatment care, which includes timely checkups and screening. Created with BioRender.com.

A precise identification of the underlying genetic cause of a disease is a critical first step in developing a therapeutic genome editing method for the disease. LRS and CRISPR-based genome editing together establish a powerful, interconnected framework for personalized precision medicine in rare diseases (Figure 3). LRS facilitates high-resolution detection of pathogenic variants, including those located in deep intronic, repetitive, or structurally complex regions inaccessible to short reads. This accurate detection directly informs gene-editing design by identifying precise mutation sites, defining breakpoints, determining allele-specific variants, and revealing potential targetable regulatory elements (Figure 3). Knowledge from LRS allows researchers to design optimal gRNAs that minimize off-target regions and precisely target pathogenic mutations. Haplotype resolution further supports allele-specific editing strategies, especially in autosomal dominant disorders where selective silencing of mutant alleles is desirable. In return, CRISPR-based editing enables functional validation of variants detected through LRS. SVs, or repeat expansions, identified through long-read technology can be modeled *in vitro* using CRISPR tools to elucidate disease mechanisms. Furthermore, correction of these mutations can also be achieved through CRISPR technology using cell lines, animal models and clinical trials. This synergy accelerates the pathway from diagnosis to therapeutic interpretation. Together, LRS and CRISPR form a unified precision-medicine pipeline (Figure 3) such that it improves diagnostic accuracy and accelerates translation toward personalized therapeutics.

## Translational, ethical, and regulatory perspectives

Although long-read sequencing (LRS) and next-generation CRISPR-based gene-editing technologies hold transformative potential for rare disease diagnosis and treatment, their translation into routine clinical practice remains constrained by interrelated technical, economic, ethical, and regulatory challenges (19, 20, 80). A notable limitation of the proposed pipeline is the current focus of gene editing tools on coding-region mutations, despite evidence that approximately 79% of rare variants contributing to narrow-sense heritability are non-coding in nature (81). Importantly, LRS has demonstrated considerable strength in detecting pathogenic variants residing in non-coding regulatory elements such as promoters, enhancers, silencers, and topologically associating domain boundaries (20). Future refinement of this pipeline should therefore incorporate gene editing strategies specifically tailored to non-coding regions, where gRNA design presents unique structural and functional challenges compared to coding-region targets (82).

From a translational perspective, the proposed LRS-guided CRISPR pipeline spans a spectrum of clinical readiness. At the diagnostic end of the pipeline, LRS, using both PacBio HiFi and ONT platforms, can detect a broad spectrum of disease-causing variants in a single assay, including SVs, copy number changes, tandem repeat expansions, phased compound heterozygous alleles, and aberrant methylation signatures that are often difficult to detect using SRS. However, its integration into routine clinical practice is still evolving (45, 83). Challenges around bioinformatics pipelines, sharing variant databases, accredited reporting workflows, and cost-effective turnaround strategies are still being standardized across centers, meaning that LRS currently occupies a transitional space between research-grade and routine clinical practice (45, 83). Once a pathogenic or likely pathogenic variant is identified, functional validation in patient-derived cells like fibroblasts and induced pluripotent stem cells can provide an important next step for establishing variant pathogenicity (84). Progression to animal models, such as precision knock-in mice models designed to recapitulate the human pathogenic variant, is feasible for preclinical phenotyping and therapeutic outcome measurement, though species-specific differences in phenotype expression and the time required for model characterization represent a meaningful translational bottleneck (84).

At the clinical end of the pipeline, CRISPR-based therapies have entered the clinical implementation phase. Ex vivo gene-editing approaches for rare hematological diseases have received regulatory approval, and the first reports of patient-specific *in vivo* gene editing for an individual rare genetic disease have been published (66, 85). Nevertheless, key barriers remain before CRISPR therapies can be broadly applied, including efficient and tissue-specific delivery, off-target risk mitigation, immunogenicity management, long-term safety, large-scale manufacturing, and regulatory pathway navigation (67, 68, 75). Taken together, the proposed pipeline is grounded in existing technological capabilities and published proof-of-concept studies. The stages of the pipeline differ substantially in their levels of clinical maturity. Consequently, this proposed pipeline should be viewed as a translational framework that outlines a potential pathway from diagnosis to therapy rather than a routine clinical workflow currently applicable to most patients with rare genetic diseases.

## Ethical deliberations: somatic vs. germline editing

Ethical considerations are deeply intertwined with these translational challenges. Somatic gene editing aimed at therapeutic intervention is generally regarded as ethically permissible when supported by compelling evidence of safety, proportionality, and clinical benefit (80). In contrast, germline genome editing raises profound ethical concerns due to its heritable nature and irreversible impact on future generations. Risks of normalizing enhancement-based applications, exacerbating social inequality, or imposing irreversible genetic changes without the consent of future individuals underscore the need for clear ethical boundaries and international governance consensus (80, 86).

Equitable access further complicates the ethical landscape. Advanced genomic diagnostics and gene-editing therapies remain resource-intensive, raising concerns that precision medicine may deepen existing global health disparities, particularly in low-resource settings (44, 86). Responsible integration, therefore requires attention not only to safety but also to distributive justice.

## Islamic ethical foundations in the gulf context

In the Gulf region, ethical deliberations surrounding gene-editing technologies frequently draw upon Islamic legal and moral traditions. Classical jurisprudence does not directly address genome editing; however, contemporary Islamic bioethical scholarship engages the issue through established juristic principles such as ḍarar (avoidance of harm) and maṣlaḍa (promotion of public interest or benefit) (87, 88).

These principles have historically allowed accommodation of biomedical innovation when it serves therapeutic and life-preserving purposes. Scholars argue that direct answers to modern genetic dilemmas are unlikely to be found in classical texts in simplistic form; rather, renewed juristic reasoning (ijtihâd) is required to develop context-sensitive ethical frameworks capable of addressing technologies as complex as genome editing (87, 88).

Contemporary discourse often distinguishes between therapeutic somatic editing, potentially permissible under principles of harm prevention and medical necessity, and germline modification, which raises deeper concerns due to intergenerational consequences and uncertainty regarding long-term effects (89). Calls have therefore emerged for the development of ḍawābiḍ (normative boundaries or regulatory standards) to ensure that gene-editing practices remain within ethically recognizable limits (90, 91).

Importantly, Islamic legal scholarship has historically demonstrated flexibility toward beneficial medical advancement, particularly when aligned with prevention of harm and realization of substantial benefit (88, 92). This adaptive tradition provides a shared normative substrate across GCC jurisdictions, even where statutory codification differs.

## Regulatory models in the gulf cooperation council

From a regulatory standpoint, governance frameworks across the Gulf region remain heterogeneous.

The United Arab Emirates currently represents the most advanced statutory model through Federal Decree-Law No. 49 of 2023 on the Regulation of the Use of the Human Genome (89). The law establishes binding requirements for informed consent, data protection, confidentiality, and safeguards against genetic discrimination, consistent with broader international bioethical standards (93). It further regulates the handling of biological samples and genomic data, creates centralized genomic governance structures, and draws a clear distinction between prohibited non-therapeutic genomic modification and conditionally permitted therapeutic gene editing subject to licensing and clinical oversight.

In contrast, other GCC states, including Saudi Arabia and Qatar, primarily rely on institutional and bioethical governance mechanisms rather than genome-specific legislative instruments. Saudi Arabia has developed large-scale genomic infrastructure through the Saudi Human Genome Program (37), with oversight exercised through the National Committee of Bioethics and research ethics regulations (94). Qatar has similarly implemented a research-centered genomic governance model through the Qatar Genome Program (36), supported by personal data protection legislation and institutional research governance frameworks (95). While these systems provide structured ethical review and research oversight, they do not yet constitute comprehensive statutory frameworks specifically regulating genome editing or germline modification.

This regulatory asymmetry reflects a broader transitional phase in regional governance: from ethics-committee–based biomedical regulation toward more formalized legislative codification.

As illustrated in Table 6, the GCC region demonstrates varying degrees of formal legislative codification, with the UAE adopting a statute-based model, while other states rely primarily on institutional and bioethical governance mechanisms.

**TABLE 6:** Comparative regulatory approaches to genomic governance in GCC states.

Country	Genome program	Dedicated genome law	Research ethics governance	Data protection law	Regulation of gene editing
UAE	UAE genome program	Yes (decree-law 49/2023)	Yes	Yes	Therapeutic permitted; enhancement prohibited
Saudi Arabia	Saudi human genome program	No standalone genome law	NCBE + IRBs	Yes (PDPL 2021)	Regulated via bioethics + SFDA
Qatar	Qatar genome program	No standalone genome law	Institutional IRBs	Yes (Law No.13/2016)	Research-regulated; no genome-specific statute
Oman/Kuwait/ Bahrain	Limited national programs	No	Research ethics committees	Yes (general data laws)	No specific gene-editing legislation

## Integrating governance into precision medicine pipelines

The successful deployment of LRS-guided CRISPR pipelines for rare disease precision medicine depends on more than technological innovation alone. Sustainable progress requires:

1. Scientific validation and clinical safety.
2. Ethical legitimacy grounded in cultural and religious norms.
3. Adaptive regulatory systems capable of responding to rapid technological change.

Embedding legal and ethical governance within translational workflows is essential to foster public trust, protect patient autonomy, and prevent misuse. In the Gulf region, harmonization across jurisdictions may become increasingly important as cross-border clinical trials, genomic data exchange, and regional therapeutic collaborations expand.

Ultimately, the integration of long-read sequencing and next-generation gene-editing technologies represents not merely a technical convergence, but a normative challenge: aligning precision medicine innovation with ethical accountability and legally coherent governance frameworks capable of safeguarding both present and future generations (89).
